# Who is going to pay the price of Covid-19? Reflections about an unequal Brazil

**DOI:** 10.1186/s12939-020-01207-2

**Published:** 2020-06-08

**Authors:** Fabiana Ribeiro, Anja Leist

**Affiliations:** 1 Institute for Research on Socio-Economic Inequality (IRSEI), University of Luxembourg, Esch-sur-Alzette, Luxembourg; 2 Centro de Matemática, Computação e Cognição, Universidade Federal do ABC, São Bernardo do Campo, Brazil

**Keywords:** COVID-19, Low-income population, Government discrepancies, COVID-19, População de baixa renda, Discrepâncias governamentais

## Abstract

The COVID-19 pandemic has caused high mortality rates among older people, and in order to avoid a healthcare system crisis, almost all countries worldwide have adopted social isolation measures to prevent the spread of the disease. However, in Brazil, a country demarcated by economic inequalities, in which approximately 25% of the population live below the poverty line, these measures will cost severe economic losses and accentuated starvation. For this reason, the underprivileged population should be immediately prioritized and well informed through good practice to avoid the virus. Since, government discrepancies in dealing with the COVID-19 outbreak leaves the population without congruent guidelines on how to react or what to believe, allowing the spread of fake news and political crises. Here, we discuss who will pay the price of the Brazilian government denying the impact of COVID-19 pandemic and suggest some measures to ensure that clear information and protection reach this population.

## Resumo

A pandemia da COVID-19 tem causado altas taxas de mortalidade entre os idosos e para evitar uma crise no sistema de saúde, quase todos os países do mundo adotaram medidas de isolamento social para evitar a disseminação da doença. Entretanto, no Brasil, um país demarcado pelas desigualdades econômicas, no qual aproximadamente 25% da população vive abaixo da linha de pobreza, essas medidas custarão severas perdas econômicas e acentuarão a fome. Por essa razão, a população desprivilegiada deve ser imediatamente priorizada e bem informada através de boas práticas para evitar o vírus. Visto que as discrepâncias governamentais no tratamento da epidemia da COVID-19 deixam a população sem diretrizes congruentes sobre como reagir ou no que acreditar, permitindo a disseminação de notícias falsas e crises políticas. Aqui, discutimos quem vai “pagar a conta” do governo brasileiro negando o impacto da pandemia da COVID-19 e sugerimos algumas medidas para garantir que proteção e informações claras cheguem a essa população.

## Introduction

We have been facing a pandemic crisis caused by COVID-19, which has produced high hospitalization rates after infection and an elevated mortality rate among the elderly aged over 60. To partially decrease public health problems, interventions relying on travel bans, social distancing, quarantine, and self-isolation have been implemented in almost all countries worldwide. These practices prevent the spread of potential infection to other individuals; nevertheless, it will cost, in the short term, severe economic damage.

The situation in Brazil is rapidly evolving as in all countries over the last months. Almost three months ago, on 25th February, Brazil confirmed the first case of COVID-19 in the state of São Paulo, brought by a wealthy patient coming from Italy, who spread the virus to other people, including her domestic servant with an age of over 60 years who would die a few days later. In the face of this scenario, several experts have already argued that COVID-19 affects people differently, in other words, according to their social class: the marginalized populations will suffer the most significant consequences [1–3].

At this moment, Brazil is considered the new global centre of the pandemics, with substantial increases in daily deaths, and mounting to more than 19,000 deaths due to COVID-19 in total. These are the possible results of late measures of quarantine implementation by governors, strongly criticized by the president. Furthermore, these numbers seem to be underestimated since testing has been carried out in approximately 3462 people per 1 million, which represents one of the lowest testing numbers in the world. It is essential to highlight that since the outbreak of the COVID-19 pandemic, the situation in Brazil can be characterized as official communication not adhering to the WHO guidelines and a series of measures that ignored the danger of the COVID-9 crisis for public health, leading to a spread of the epidemic across the country.

Brazil is characterized by increased inequalities, such as a large number of low-income people and a small elite that controls economic and political powers [4]. Poverty in Brazil affects mainly blacks and browns who compose 72.7% of low-income people, of which over half are women. These people live in crowded living conditions and with limited possibilities to follow hygiene- and social distancing related recommendations in many urban communities, which makes a significant, hardly containable spread of the disease very likely to happen. Allied to this, a large part of the people over 60 years old, cannot follow the quarantine recommendations since they have to work to, with their income, still help a large number of family members as a consequence of the weak system of pensions which favours the non-poor [5].

Moreover, 6.9% of the Brazilian population is illiterate, 30% of the Brazilians between the age of 15 to 64 years old are functionally illiterate people [6], 26.9% have 11 years of formal education, and only 16.5% have completed undergraduate education [7], which means that a large part of the population is unable to interpret information related to COVID-19, especially when conflicting messages are disseminated.

Another important point regarding inequality in Brazil is related to the public health system that covers healthcare of approximately 80% of the population [7], of which many people who cannot afford to pay for private healthcare. In other words, Brazil has a public health system that can rapidly collapse if measures to prevent the spread of the virus are not followed. It has been shown in Brazil that low schooling and consequently low purchasing power combined with a weak health system is aggravating the development of chronic health problems in the population: Beltrán-Sánchez and Andrade [8] noticed that Brazilians without formal education had higher levels of diabetes, hypertension, and heart disease as a result of higher prevalence of obesity compared to those with some formal education, especially among women.

Congruently, with a higher number of adults presenting chronic diseases, the impact of COVID-19, which has caused a high number of deaths among older people in developed countries, in a developing country like Brazil, the possible victims will also be younger ones. This trend has already been observed, as 30% of the reported deaths were from patients under the age of 60 [9].

### Who is going to pay the price of COVID-19 in Brazil?

The marginalized groups that support the elite of Brazilian society will be the potential victims of COVID-19, which represent informal workers or those in essential services [10]. Although more men are dying at this moment from COVID-19 around the world, outcomes of COVID-19 seem to be associated with health comorbidities and also by social inequalities [11]. Given that women in Brazil are those who integrate the most significant numbers of informal jobs [7] it is possible to suppose that more women will be affected by COVID-19 when compared to countries with greater gender equality.

Furthermore, the numbers of the Ministry of Health [9] have been showing that, when considering hospital admissions, the percentage of white persons hospitalized for COVID-19 is higher than the rates of black and brown persons. However, about 54.8% of deaths are of the latter, which are overwhelmingly users of the public health system.

As urgent measures to ensure that the situation does not get even worse, the government must promote, with information based on scientific evidence, public health campaigns with congruent and clear guidelines across the country using easy language (e.g. distributing flyers and providing masks), so its population can understand how to act.

Furthermore, the population of Brazil needs to be tested, and contacts of cases need to be traced, to provide precise data on the pandemic and stop further spread. Without testing and tracing, there can be no effective healthcare response.

## Data Availability

Not applicable.
